# The Path of Most Resistance: A Rare Instance of Metastatic Pancreatic Adenocarcinoma Identified Within Skeletal Muscle

**DOI:** 10.7759/cureus.13947

**Published:** 2021-03-17

**Authors:** Caitlin E Harmon, Sanjay P Lamsal, Taylor S Harmon, Khaled Mohamed, Travis E Meyer

**Affiliations:** 1 Radiology, University of the Incarnate Word School of Osteopathic Medicine, San Antonio, USA; 2 Radiology, University of Florida College of Medicine, Jacksonville, USA; 3 Pathology, University of Florida College of Medicine, Jacksonville, USA

**Keywords:** pancreatic adenocarcinoma, skeletal muscle metastasis, interventional radiology, carbohydrate antigen 19-9, core needle biopsy, carcinoembryonic antigen, positron emission tomography, computed tomography, desmoplastic microenvironment, whipple procedure

## Abstract

Soft tissue neoplastic metastases, specifically to the skeletal muscle, are uncommon in comparison to metastases within the abdomen, thorax, or intracranial regions. Specifically, pancreatic adenocarcinoma with skeletal muscle metastasis is a rare clinical phenomenon and is hardly reported. There is a high mortality rate after the diagnosis of metastatic pancreatic adenocarcinoma, attributed to inadequate screening and advanced staging upon incidental discovery. Rarely, metastatic lesions manifest in the skeletal muscle and are hardly documented. Some of the factors that deter skeletal muscle tumor implantation include the deficiency of skeletal muscle mediators and genetic makeup of the primary tumor. Surgical resection of pancreatic adenocarcinoma with adjuvant chemotherapy demonstrates the best prognosis; however, surgical management is usually limited to patients without known metastatic disease. Alternative treatment options such as chemotherapy and radiotherapy are used in the palliative care setting. Here, we present the case of a patient with previously diagnosed and treated pancreatic adenocarcinoma in remission, with biopsy-proven metastases isolated within the skeletal muscle.

## Introduction

Pancreatic adenocarcinoma is the most common variant of pancreatic carcinoma [1]. Pancreatic carcinoma commonly presents in advanced stages with limited therapeutic options upon diagnosis, and is categorized last in cancer prognostic outcomes [2]. Pancreatic adenocarcinoma has a five-year survival rate of 2-9%, being the fourth cause of cancer-related death in the United States [2]. The risk factors are complex and multifactorial, including advanced age, African American descent, smoking, diabetes mellitus, chronic pancreatitis, and a history of familial pancreatic cancer [3].

Inherited susceptibility of pancreatic carcinoma originates from germline mutations of the *BRCA*, *KRAS*, and *PALB2* genes [3]. Ongoing studies harbor the interplay between these genetic alterations and pancreatic adenocarcinoma precursor lesions that ultimately determine the aggressive nature of the disease and the subsequent poor prognosis [2]. Although the current literature defines the mechanisms of tumorigenesis and invasion, the molecular pathways that drive site-specific colonization of pancreatic adenocarcinoma remain unknown. The common sites of pancreatic adenocarcinoma metastases are the liver, lungs, peritoneum, and bone, with less than 1% of these metastases discovered in intramuscular tissue [4,5]. Additionally, metastasis of any neoplasm to the skeletal muscle has a prevalence of 0.33%, with the most common primary malignancy originating from the lung [6]. Here, we present the case of a patient with previously diagnosed and treated pancreatic adenocarcinoma in remission, with biopsy-proven metastases isolated within the skeletal muscle.

## Case presentation

A 62-year-old female with a past medical history of diabetes and pancreatic adenocarcinoma, status post-pancreaticoduodenectomy (Whipple procedure) and neoadjuvant chemotherapy with leucovorin, fluorouracil, irinotecan hydrochloride, and oxaliplatin (FOLFIRINOX combination), presented to the emergency department with generalized pain along her left upper back and neck. The patient described a painful palpable lesion located along her left neck and superior back that had progressively worsened in the last few months, despite pain management with oxycodone/acetaminophen and lidocaine patches.

The patient reported close surveillance with the oncology service, and according to oncology documentation, was in remission of her pancreatic adenocarcinoma since completing chemotherapy five years earlier. At the time of treatment, the patient completed four rounds of FOLFIRINOX therapy in distinct intervals of two weeks. Approximately six weeks following her final chemotherapy treatment, she underwent pylorus-preserving pancreaticoduodenectomy and cholecystectomy. Forty-eight days post-operatively, the patient completed six months of adjuvant gemcitabine therapy. Surveillance positron emission tomography/computed tomography (PET/CT) imaging was performed every six months thereafter.

**Figure 1:**
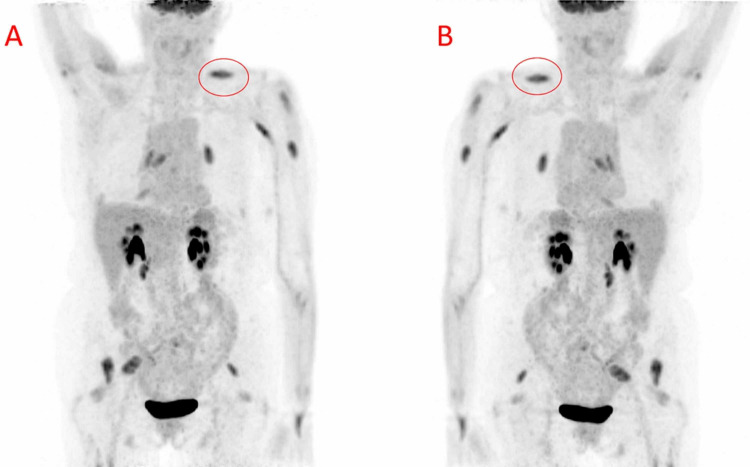
Maximum intensity projection PET images demonstrating multiple skeletal musculature metastases. Maximum intensity projection anterior (A) and posterior (B) PET demonstrates fludeoxyglucose F-18 avid metastatic lesions diffused throughout the skeletal musculature. There is no evidence of fludeoxyglucose F-18 avidity within the region of the pancreaticoduodenectomy bed to suggest residual or local recurrence. The metabolically active left trapezius lesion (red circle) was targeted with ultrasound-guided core biopsy, and subsequently verified by histopathological analysis. PET, positron emission tomography

The patient was discharged from the emergency department and scheduled for a close follow-up outpatient PET/CT in the setting of her new symptoms and to observe for recurrent metastatic disease. On imaging, metabolically active lesions within the left trapezius, bilateral deltoids, left pectoralis major, left triceps, paraspinal musculature, bilateral piriformis muscles, and right iliopsoas were identified (Figure 1).

**Figure 2:**
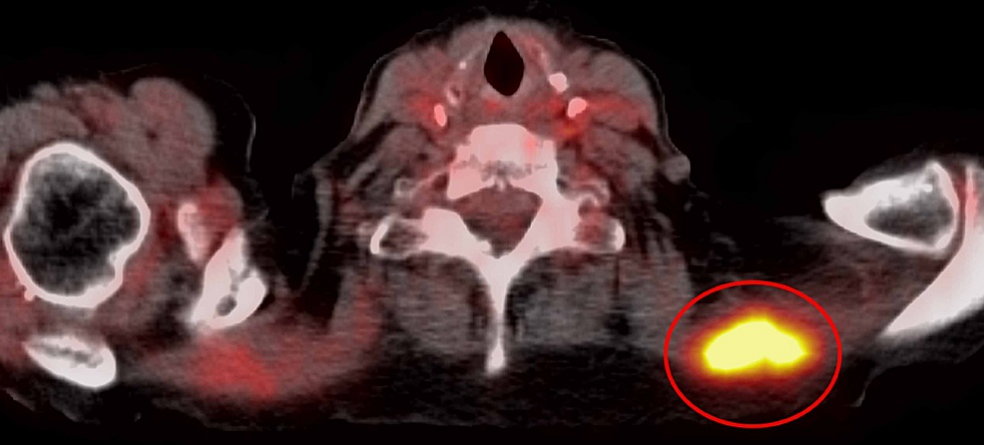
Biopsied left trapezius lesion identified on PET/CT. FDG PET/CT reconstructed images demonstrate the FDG avid left trapezius lesion with a maximum standardized uptake value of 7.7 (red circle). This lesion corresponds to biopsy-proven metastatic pancreatic adenocarcinoma by histopathological analysis. PET/CT, positron emission tomography/computed tomography; FDG, fludeoxyglucose F-18

The interventional radiology service was consulted for an ultrasound-guided core biopsy of the most accessible fludeoxyglucose F-18 (FDG) avid lesion, which was within the left trapezius (Figure 2).

**Figure 3:**
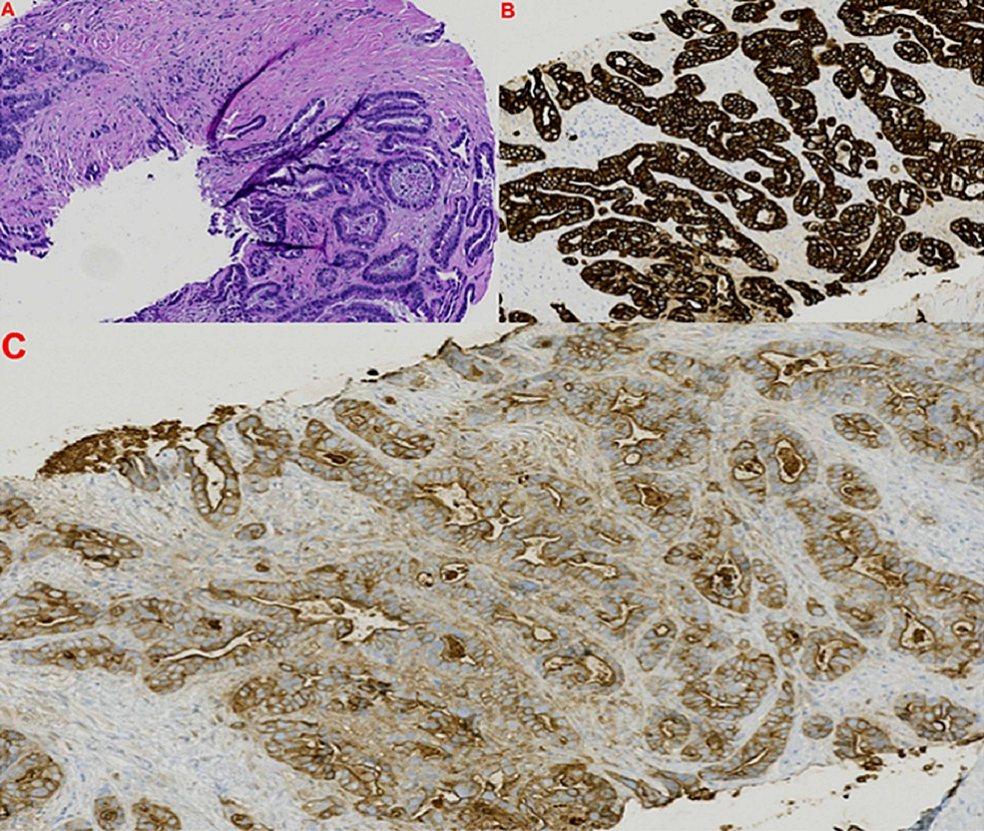
Core biopsy sample histopathological analysis demonstrating metastatic pancreatic adenocarcinoma within skeletal muscle. Hematoxylin and eosin preparation at 100× magnification (A) demonstrates microscopic view of well-differentiated adenocarcinoma mixed with skeletal muscle. Cytokeratin 7 positive staining shows strong diffuse cytoplasmic and membranous staining at 100× magnification (B). Carbohydrate antigen 19-9 positive staining demonstrates cytoplasmic staining at 100× magnification (C).

Under ultrasound guidance, an 18-gauge core biopsy device was positioned and deployed within the left trapezius lesion. A total of three adequate core biopsy specimens were obtained, and histopathological analysis was performed (Figure 3).

The final histopathology analysis confirmed metastatic pancreatic adenocarcinoma. Upon histopathological verification of disseminated skeletal muscle metastases (SMM), chemotherapy was scheduled to be reinstated. Unfortunately, the patient passed away before subsequent treatment.

## Discussion

Skeletal muscle encompasses half of the total human body mass and contains adequate vascular supply that would seemingly be optimal for tumor growth. However, neoplastic metastasis to skeletal muscle remains an uncommon occurrence [7]. Some evidence suggests complex crosstalk between the primary tumor and the surrounding desmoplastic microenvironment of distant metastatic sites, which rarely occurs in skeletal muscle [8]. As discussed, pancreatic adenocarcinoma metastases are most commonly found in the liver, lungs, peritoneum, and bone, with less than 1% discovered in intramuscular tissue [4,5]. Furthermore, SMM from any primary tumor is a rare phenomenon, with a prevalence of less than 1% [9].

There are several well-studied explanations that may describe why skeletal muscle is uninhabitable by neoplastic metastases, emphasizing local cellular mediators that biomechanically compromise the environment required for tumor colonization. These native immune-mediated entities are composed of anti-cancer cytokines, lymphocyte infiltration factors, and proteolytic enzymes [7]. Considering the patient’s medical history of diabetes, intramuscular colonization may have been accentuated by variations in cell mediators and growth factor imbalances, favoring metastatic implantation.

There has been documented evidence demonstrating the onset of certain primary tumor metastasis by carcinogenic growth factors that originate from early genetic alterations of precursor lesions [1]. In descending order, pulmonary (25.1%), gastrointestinal (21.0%), and urological (13.2%) tumors are the most commonly documented primary neoplasms that metastasize to skeletal muscle [10]. A striking 1% of these SMM originate from primary tumors of the pancreas [10,11]. SMM can appear in any striated musculature; however, most localize in the paravertebral skeletal muscle and lower truncal region [10]. This is congruent with the preceding case, where SMM were demonstrated within the paravertebral musculature and lower trunk, as well as uniquely within the left trapezius, deltoid, and triceps. Documented evidence of SMM within the upper extremity musculature is rare and is seen in less than 5% of the cases [12]. Metastatic invasion depends on the location of the primary tumor and the surrounding vascular points of entry [13]. Pancreatic adenocarcinoma spreads hematogenously through the celiac axis or portal veins, with colonization most often observed in the peritoneum (42.3%), liver (41%), and lungs (13.9%) [4]. Considering the rarity of SMM, the mechanism for vascular upper extremity dissemination and intramuscular colonization in our case remains unknown.

The clinical presentation of SMM varies in each individual patient. Although our patient was symptomatic upon presentation, the majority of documented SMM are asymptomatic and incidentally identified on routine imaging [12]. Therefore, routine surveillance imaging, even when patients are considered in remission, is important when monitoring for current developing metastases such as SMM [14]. FDG PET/CT was appropriately utilized in the preceding case while the patient was in remission, though multiple skeletal metastases were found as a result of the patient’s symptoms rather than incidentally [15]. Interestingly, FDG avid lesions were isolated within the skeletal muscle, rather than other more common areas where pancreatic adenocarcinoma is known to metastasize. Additionally, the post-operative pancreatic surgical bed remained free of metastases. The absence of locoregional recurrence and common metastatic sites was unique and has hardly been reported.

Soft tissue neoplastic metastasis is indicative of advanced disease, limiting further treatment prognosis. Radiation therapy, limited surgical management, and opioid analgesics are utilized in advanced disease and in the palliative care setting [13]. Surgical resection (Whipple procedure) and adjuvant chemotherapy have the best patient prognosis for primary pancreatic adenocarcinoma that has not metastasized to distant viscera [3]. However, adenocarcinoma is usually discovered in advanced stages, rendering 80-90% of patients ineligible for these treatments [16]. Therefore, alternative locoregional therapies for SMM are currently being investigated [17]. Clinicians should be aware of the aggressive metastatic potential of pancreatic adenocarcinoma. FDG PET/CT and tumor markers such as carbohydrate antigen 19-9 and carcinoembryonic antigen should be considered invaluable tools in the clinicians armamentarium for metastatic surveillance.

## Conclusions

Primary tumor metastasis to skeletal muscle is rare and hardly documented due to the various intrinsic factors that make striated musculature uninhabitable. Furthermore, the metastasis of pancreatic adenocarcinoma to skeletal muscle has been documented in less than 1% of all metastatic lesions. We presented the case of a patient with a previously diagnosed and optimally treated pancreatic adenocarcinoma in remission, with the current development of biopsy-proven metastases isolated within the skeletal muscle.
